# Editorial: Organic-Based Foliar Biostimulation and Nutrition in Plants

**DOI:** 10.3389/fpls.2015.01131

**Published:** 2016-01-07

**Authors:** Jose M. Garcia-Mina, Ebrahim Hadavi

**Affiliations:** ^1^Faculty of Sciences, Agricultural Chemistry and Biology Group - CMI Roullier, Department of Environmental Biology, University of NavarraPamplona, Spain; ^2^Horticulture Department, Karaj Branch, Islamic Azad UniversityKaraj, Iran

**Keywords:** biostimulants, foliar spray, foliar nutrition, cuticle, plant biostimulant

Biostimulants, are a wide range of organic or synthetic based products which can enhance the plant performance specially in presence of biotic or abiotic stresses (du Jardin, 2015). Usually, the mode of action of observed responses are not well understood. However, there is an agreement that the response should not be due to presence of essential mineral elements, known plant hormones, or disease suppressive molecules (Brown and Saa). Nowadays the environmental concerns and residuals on edible product is a matter of awareness that is going to put more limitations on use of synthetic foliar formulations in future. The high price of many commercial formulations and dependence on non-renewable resources are other constrains. On the other hand, the increased interest in sustainable agriculture favors the studies on more environmental friendly organic-based materials, which are more readily available (Russo and Berlyn, 1991).

In current research topic we focused on those known biostimulants that are produced from naturally occurring substances. This group of biostimulants while have the advantage of being less costly to farmer but are not defined in composition and could contain a vast diversity of molecules from which, a few have known mode of actions. Our focus was to start an initiative to enable us to understand this complexity in future. The cumulative information in this area could help to reach a fundamental approach in formulation of foliar sprays based on natural ingredients. To make this happen there is a need to include other known types of biostimulants in a stepwise manner that would help to create a clearer picture from the mode of action of this group of biostimulants in future.

The presenting authors were invited based on a record of work in this field and are mostly among prominent researchers in the field. The articles were critically reviewed by outstanding researchers in the field to create a sound base for further studies in this area of research.

The presented articles are very well summarized in a Mini-Review article by Brown and Saa that provided an insight to the gathered knowledge by the research topic that we suggest reading it prior to the published articles. We hope that by presented insights, further works could reveal more from the secrets behind the action of biostimulants. This could let us leave current try and error phase and enter to formulation stage of organic-based biostimulants in future.

## Author contributions

The text is written by EH and reviewed by JG.

### Conflict of interest statement

The authors declare that the research was conducted in the absence of any commercial or financial relationships that could be construed as a potential conflict of interest.
